# Effects of Organic Substrate Concentrations on Biological Mn Oxidation and Reduction under Aerobic Conditions

**DOI:** 10.1264/jsme2.ME26046

**Published:** 2026-09-12

**Authors:** Yangbo Chen, Mizuki Yoshimura, Hiromi Kambara, Ahmad Shoiful, Shuji Matsushita, Tomonori Kindaichi, Yoshiteru Aoi, Akiyoshi Ohashi

**Affiliations:** 1 Department of Civil and Environmental Engineering, Graduate School of Advanced Science and Engineering, Hiroshima University, 1–4–1, Kagamiyama, Higashi-Hiroshima, Hiroshima 739–8527, Japan; 2 Biomanufacturing Process Research Center, National Institute of Advanced Industrial Science and Technology (AIST), 2–17–2–1, Tsukisamu-Higashi, Toyohira-ku, Sapporo, Hokkaido 062–8517, Japan; 3 Research Center for Environmental and Clean Technology, National Research and Innovation Agency, Geostech Building 820, B.J. Habibie Science and Technology Park, Serpong, Tangerang Selatan 15314, Indonesia; 4 Agricultural Technology Research Center, Hiroshima Prefectural Technology Research Institute, 6869, Hara, Hachihonmatsu, Higashi-Hiroshima, Hiroshima 739–0151, Japan; 5 Program of Biotechnology, Graduate School of Integrated Sciences for Life, Hiroshima University, 1–3–1, Kagamiyama, Higashi-Hiroshima, Hiroshima 739–8530, Japan

**Keywords:** aerobic Mn(IV) reduction, biological manganese oxidation, manganese-oxidizing bacteria, substrate inhibition

## Abstract

Biological Mn oxidation mediated by manganese-oxidizing bacteria (MnOB) plays a central role in Mn cycling in natural and engineered systems. Although most MnOB are heterotrophic and frequently associated with oligotrophic environments, the quantitative relationship between organic substrate concentrations and Mn oxidation activity remains unclear. We herein systematically evaluated the effects of organic substrate concentrations on MnOB activity using a down-flow hanging sponge (DHS) reactor (0–1,000 mg COD L^–1^), batch experiments with an enriched biomass, and the pure MnOB strain *Pseudomonas resinovorans* MO-1 (0–2,000 mg COD L^–1^). Mn(II) oxidation was favored at organic substrate concentrations below approximately 50 mg COD L^–1^, whereas concentrations around 100 mg COD L^–1^ were more favorable for MnOB growth. In contrast, elevated organic substrate concentrations induced substrate inhibition and suppressed Mn(II) oxidation. At high organic substrate concentrations (≥1,000 mg COD L^–1^), net Mn(IV) reduction by *P. resinovorans* MO-1 occurred even under aerobic conditions. An exploratory transcriptomic comparison showed patterns that were broadly consistent with the observed shifts in Mn redox behavior. Collectively, these results define an operational organic substrate concentration window for stable MnOB enrichment and efficient Mn(II) oxidation, and indicate the potential of excessive organic substrate concentrations to shift MnOB activity toward net Mn(IV) reduction under aerobic conditions.

Heavy metal contamination in wastewater and groundwater remains a persistent environmental concern (Mustapha *et al.*, 2026). In this context, biogenic manganese oxides (bio-MnO_x_) produced by manganese-oxidizing bacteria (MnOB) have attracted considerable attention as promising materials for heavy metal removal due to their highly reactive surfaces as well as their ability to adsorb and oxidize harmful metals (Tebo *et al.*, 2004; Li *et al.*, 2024). However, their practical application remains limited because stable MnOB enrichment and continuous bio-MnO_x_ production are still difficult to achieve in engineered systems.

Under circumneutral conditions, biological Mn(II) oxidation is considered a major pathway in many natural environments (Tebo *et al.*, 2004). Bacteria capable of oxidizing Mn(II) to bio-MnO_x_ are collectively referred to as MnOB. Notably, MnOB have frequently been detected in oligotrophic environments (Tebo *et al.*, 2005; Miyata *et al.*, 2024). Although chemolithoautotrophic MnOB capable of utilizing CO_2_ as a carbon source have been identified (Yu and Leadbetter, 2020), the majority of characterized MnOB are heterotrophs and are frequently associated with low-organic environments. Consistent with this ecological pattern, MnOB enrichment in engineered systems has been achieved under low organic substrate conditions or through syntrophic associations with nitrifying and methanotrophic bacteria, in which MnOB utilize secondary metabolites produced by partner organisms (Cao *et al.*, 2015; Matsushita *et al.*, 2018a; Shoiful *et al.*, 2020). These findings suggest that many MnOB are adapted to environments with limited organic substrate availability, which may partly explain why stable MnOB enrichment and continuous bio-MnO_x_ production remain difficult in engineered systems.

Although the mechanisms underlying Mn(II) oxidation by MnOB remain unclear, multicopper oxidases (MCOs) and animal heme peroxidases (AHPs) are widely recognized as key contributors (Geszvain *et al.*, 2012). In model MnOB, such as *Bacillus* SG-1, *Pseudomonas putida* GB-1, and *P. resinovorans* MO-1, genes within the MCO cluster (*cotA*, *moxA*, *mnxG*, and *mcoA*), as well as the AHP gene (*mopA*), have been shown to directly catalyze Mn(II) oxidation (Anderson *et al.*, 2009; Geszvain *et al.*, 2013; 2016; Matsushita *et al.*, 2018b). Mn(II) oxidation was also shown to be affected by flagellar-associated genes (Geszvain *et al.*, 2011). More recently, catalase–peroxidase (*KatG*) was identified as a novel Mn(II)-oxidizing enzyme in *Salinicola tamaricis* (Zhao *et al.*, 2023). In addition to these direct enzymatic pathways, indirect Mn(II) oxidation mediated by reactive oxygen species or secondary metabolites has been suggested in some MnOB (Jofré *et al.*, 2021).

In contrast to Mn(II) oxidation, Mn oxides may also function as electron acceptors under anaerobic conditions. In such cases, biological Mn(IV) reduction has classically been attributed to metal-reducing bacteria, such as *Geobacter* and *Shewanella*, which use organic substrates as electron donors and transfer electrons to MnO_2_ via extracellular electron transfer pathways (Lovley *et al.*, 2004; Logan, 2009). However, Gounot (1994) demonstrated that MnOB within the genera *Bacillus* and *Pseudomonas* were capable of Mn(II) oxidation under aerobic conditions and Mn(IV) reduction under anaerobic conditions. Notably, Mn(IV) reduction has been observed during prolonged incubations under initially aerobic conditions. For example, *Bacillus pumilus* reduced MnO_2_ under aerobic conditions after an extended incubation (Cerrato *et al.*, 2010), and similar transitions from Mn(II) oxidation to Mn(IV) reduction were detected in the presence of added organic carbon (Sujith *et al.*, 2014). Collectively, these findings indicate that Mn redox activity is dynamic and may shift depending on environmental factors, particularly organic substrate availability and the redox state.

Given this dynamic behavior, organic substrate concentrations may be a particularly important factor affecting MnOB activity because the majority of characterized MnOB are heterotrophic. Temperature, pH, oxygen availability, and Mn(II) concentrations are also known to affect MnOB activity (Tani *et al.*, 2003; Shoiful *et al.*, 2020; Cai *et al.*, 2023); however, the quantitative relationship between organic substrate concentrations and Mn(II) oxidation activity remains unclear. The concentration range over which organic substrates promote MnOB growth, inhibit Mn(II) oxidation, or even shift Mn redox behavior toward Mn(IV) reduction has yet to be systematically evaluated.

Therefore, the present study investigated the effects of organic substrate concentrations on MnOB activity and growth using a stepwise approach. MnOB were first enriched in a down-flow hanging sponge (DHS) reactor under low organic substrate conditions. After stable Mn(II) oxidation was established, organic substrate concentrations were varied to evaluate their effects on Mn(II) oxidation in the reactor (0–1,000 mg COD L^–1^). Batch experiments using a reactor-enriched biomass were then conducted to test whether the reactor-observed behavior was reproducible under batch conditions. Finally, batch experiments with the pure MnOB strain *P. resinovorans* MO-1 were performed to confirm the direct effects of organic substrate concentrations on MnOB activity, with the tested range extended to 0–2,000 mg COD L^–1^. In addition, a transcriptomic anal­ysis of *P. resinovorans* MO-1 was conducted to compare gene expression patterns under low and high organic substrate conditions.

## Materials and Methods

### Reactor configuration and operation

A DHS reactor was used to enrich MnOB and assess the effects of organic substrate concentrations on Mn(II) oxidation (Fig. 1). The reactor consisted of a vertical acrylic column (75 cm in height with an inner diameter of 5 cm) containing twenty polyurethane sponge cubes (1×1×1 cm^3^ each; total sponge volume, 20 cm^3^) suspended diagonally in series on a nylon string. The sponge cubes were inoculated by repeated squeezing and soaking in a mixed suspension of activated sludge and abiotic MnO_2_ (100 g L^–1^; Kishida Chemical). The activated sludge was obtained from the aeration tank of a municipal wastewater treatment plant in Higashi-Hiroshima, Japan. Prior to the inoculation, abiotic MnO_2_ was pre-equilibrated with Mn(II) to minimize abiotic Mn(II) adsorption during reactor operation (Shoiful *et al.*, 2024). MnO_2_ was included during the inoculation to promote MnOB enrichment (Matsushita *et al.*, 2020).

The reactor was operated at 25°C in the dark, with the substrate being continuously supplied from the top of the column. The operation consisted of three phases: Phase 1, the enrichment of MnOB under low organic substrate conditions; Phase 2, the establishment of stable Mn(II) oxidation; Phase 3, an evaluation of the effects of increasing organic substrate concentrations on reactor performance. The hydraulic retention time (HRT), calculated based on the total sponge volume (20 cm^3^), was initially set at 6 h during Phase 1 and gradually reduced to 0.5 h after complete Mn(II) removal was achieved. In Phases 2 and 3, HRT was maintained at 0.5 h. The effluent was recirculated at a ratio of 9:1 relative to the influent flow rate. Aerobic conditions were maintained by supplying air from the top of the reactor column. During Phase 3, organic substrate concentrations were varied stepwise, with each loading condition being applied for 24 h. After each high-loading test, the influent organic substrate concentration was restored to <50 mg COD L^–1^ to return the reactor to the low-organic operating conditions established in Phase 2 before the next loading step. During this phase, the gas supply was switched from air to pure oxygen to ensure sufficient aerobic conditions within the sponge cubes. Detailed operational conditions are summarized in Table 1.

### Substrate composition

The organic substrate consisted of K-medium prepared with polypeptone and yeast extract at a mass ratio of 4:1 (Matsushita *et al.*, 2020; Shoiful *et al.*, 2020, 2024). Organic substrate concentrations were adjusted between 50 and 1,000 mg COD L^–1^ and prepared in a 5-L feed tank. A second 5-L tank was used to prepare a Mn(II) solution containing essential mineral components. The organic substrate solution and the Mn(II)-mineral solution were stored separately to prevent premature interactions between organic compounds and dissolved metal species before entering the reactor. Detailed information on the substrate composition and analytical methods is provided in the Supplementary Information.

### Batch experiments with an enriched biomass

Batch experiments were conducted to independently assess the effects of organic substrate concentrations on MnOB activity using the biomass enriched in the DHS reactor. The biomass was harvested on day 160 by squeezing and washing all sponge cubes with fresh substrate to detach attached cells. The recovered biomass suspension was combined and adjusted to 200 mL. Aliquots of 20 mL were transferred into 100-mL Erlenmeyer flasks containing 80 mL of fresh substrate, corresponding to a 50-fold dilution of the reactor biomass suspension. Substrates contained 5 mg Mn(II) L^–1^ and essential minerals, with an organic substrate concentration of 50, 100, 200, or 400 mg COD L^–1^. Flasks were loosely covered with aluminum foil and continuously supplied with air using an air pump throughout the 24-h incubation at 25°C. Samples were collected at 0, 6, 12, and 24 h for Mn(II) and COD anal­yses.

### Batch experiments using P. resinovorans MO-1

*P. resinovorans* MO-1 was isolated in our laboratory and subsequently confirmed to exhibit Mn(II)-oxidizing activity (Matsushita *et al.*, 2018b). The strain is currently maintained in our laboratory and has been deposited in the NBRC culture collection (NBRC 113413). Regarding its experimental use, *P. resinovorans* MO-1 was cultivated at 25°C with orbital shaking at 100 rpm in growth medium containing 1 g L^–1^ peptone and 0.5 g L^–1^ yeast extract. After cultivation, 100 mL of the culture was centrifuged at 5,000 rpm for 5 min, and the pellet was transferred into 300-mL Erlenmeyer flasks containing the substrate. Organic substrate concentrations were adjusted to 50, 100, 200, 300, 400, 1,000, and 2,000 mg COD L^–1^. Mn(II) was supplied at 5 mg L^–1^ together with essential minerals. The total working volume was adjusted to 300 mL. In experiments at 1,000 and 2,000 mg COD L^–1^, MnO_2_ (1 g) was additionally added to assess Mn(IV) reduction under high organic substrate conditions. Flasks were loosely covered with aluminum foil and continuously supplied with air using an air pump throughout the incubation. Experiments at 50–400 mg COD L^–1^ were performed for 24 h with sampling at 0, 6, 12, and 24 h. Experiments at 1,000 and 2,000 mg COD L^–1^ were conducted for 3 d with daily sampling.

The biomass enriched in the DHS reactor was collected on day 160 for a microbial community anal­ysis. In a gene expression anal­ysis, Mn(II)-oxidizing and Mn(IV)-reducing states under aerobic conditions were established using *P. resinovorans MO-1*. Detailed methods for the microbial community anal­ysis and transcriptomic anal­ysis are provided in the Supplementary Information.

The 16S rRNA gene amplicon sequencing data obtained from DHS reactor enrichments and the transcriptomic data for *P. resinovorans* MO-1 have been deposited in the DDBJ/EMBL/GenBank database under accession number PRJDB40482.

## Results

### Reactor performance

During Phase 1, the reactor was initially operated with an influent Mn(II) concentration of 8 mg L^–1^, under which Mn(II) was not completely removed (Fig. 2). Since residual Mn(II) has been reported to inhibit the activity of MnOB (Shoiful *et al.*, 2024), the influent Mn(II) concentration was subsequently reduced to 5 mg L^–1^. Under this condition, complete Mn(II) removal was consistently achieved. HRT was then shortened stepwise to 0.5 h, and the Mn removal rate progressively increased, reaching approximately 0.20 kg Mn(II) m^–3^ d^–1^. Throughout Phase 1, the influent COD concentration was maintained below 50 mg L^–1^. More than half of influent COD was removed, and the COD removal rate gradually increased, reaching approximately 1.1 kg COD m^–3^ d^–1^. These results indicate that MnOB enrichment and biological Mn(II) oxidation were established within 41 d under low organic substrate conditions.

In Phase 2, operating conditions were unchanged and maintained for 51 d. Mn(II) and COD removal rates both remained stable, indicating that MnOB activity was sustained during this period. Based on the stable reactor performance observed in Phase 2, Phase 3 was conducted to investigate the effects of the influent COD concentration on Mn(II) oxidation. The influent COD concentration was increased stepwise from 100 to 1,000 mg L^–1^. Each high COD loading was applied only for a short period of 1 d, after which the influent COD concentration was returned to below 50 mg L^–1^, corresponding to the same conditions as those in Phase 2. This operational strategy was employed to minimize long-term changes in the MnOB community and to avoid the irreversible inhibition of Mn(II) oxidation. For example, increasing the influent COD concentration to 100 mg L^–1^ resulted in a slight decrease in the Mn(II) removal rate, while COD removal increased. When the influent COD concentration was returned to below 50 mg L^–1^, Mn(II) and COD removal rates recovered to similar levels to those observed in Phase 2 (Fig. 2). This sequence of increasing the influent COD concentration followed by restoration to the original condition was repeated multiple times. With further increases in the influent COD concentration to 200, 300, 500, and 1,000 mg L^–1^, Mn(II) removal and COD removal both exhibited marked changes.

To clarify the effects of the influent COD concentration on Mn(II) oxidation and COD removal, these relationships are presented in Fig. 3. A clear correlation was observed between Mn(II) removal and the influent COD concentration, indicating a decline in MnOB activity at elevated organic substrate concentrations (Fig. 3A). In contrast, COD removal increased with increasing influent COD concentrations up to approximately 150 mg L^–1^, at which point it peaked and subsequently declined (Fig. 3B). In general, the relationship between the substrate removal rate and substrate concentration is represented by Michaelis–Menten kinetics. However, the COD removal rate in the present study decreased after reaching its peak, suggesting the occurrence of substrate inhibition at high organic concentrations. Therefore, these results demonstrate that elevated organic substrate concentrations inhibited MnOB activity and suppressed Mn(II) oxidation.

### Batch experiments with MnOB enriched in the reactor

To confirm the effects of organic substrate concentrations on MnOB activity observed in the reactor, batch experiments were conducted using the biomass collected from the reactor at four initial COD concentrations (50, 100, 200, and 400 mg L^–1^). Changes in Mn(II) and COD concentrations were monitored over a 24-h period. Mn(II) and COD removal rates in the batch experiments were evaluated from concentration changes (Fig. S1), and then converted to units normalized to the biomass concentration in the reactor sponges, which allowed for direct comparisons with reactor performance. The Mn(II) and COD removal rates obtained from the batch experiments exhibited response patterns that were consistent with those observed in the reactor (Fig. 3), indicating strong correspondence between batch and reactor results. These results support elevated organic substrate concentrations being associated with reduced MnOB activity and substrate inhibition under high organic loading conditions.

### Batch experiments with *P. resinovorans* MO-1

Although experiments with the enriched biomass confirmed the effects of organic substrate concentrations on MnOB activity, it was not possible to exclude the contribution of microbial community interactions. To directly evaluate the effects of organic substrate concentrations on MnOB activity, batch experiments were performed using a pure culture. *P. resinovorans* MO-1 was tested under a range of initial COD concentrations corresponding to those used in the reactor and enriched-biomass batch experiments (Fig. S2). In addition, to investigate whether high organic substrate concentrations induced Mn reduction, supplementary batch tests were performed at higher initial COD concentrations of 1,000 and 2,000 mg L^–1^ with the addition of 5 mg Mn(II) L^–1^ and 1 g MnO_2_.

At low to moderate organic substrate concentrations (≤400 mg COD L^–1^), pure-culture batch experiments exhibited similar Mn(II) oxidation and COD removal patterns to those observed in both the reactor and enriched-biomass batch experiments (Fig. 4). The close agreement among reactor performance, enriched-biomass batch tests, and pure-culture experiments indicates that the inhibition observed at elevated organic substrate concentrations reflected the inherent physiological characteristics of MnOB rather than system-specific phenomena.

At high organic substrate concentrations (≥1,000 mg COD L^–1^), Mn(II) concentrations increased as MnO_2_ was reduced to Mn(II) even under aerobic conditions, indicating a reversal in the net Mn redox direction mediated by *P. resinovorans* MO-1. This result showed that an excessive amount of an organic substrate may shift MnOB metabolism from Mn(II) oxidation toward Mn(IV) reduction. Accordingly, Mn reduction may also have occurred under high organic substrate concentrations in the reactor.

Overall, these results demonstrate that organic substrate concentrations play a central role in regulating MnOB activity. Low organic substrate concentrations favor Mn(II) oxidation, whereas moderate organic substrate concentrations lead to substrate inhibition, and high organic substrate concentrations may induce Mn reduction mediated by MnOB even under aerobic conditions.

### Microbial community anal­ysis

The microbial community was analyzed using biomass samples collected at the end of reactor operation. Amplicon sequencing yielded a total of 193 amplicon sequence variants (ASVs). Taxa with relative abundances greater than 1% are shown in Table S1. At the phylum level, *Proteobacteria*, *Bacteroidetes*, and *Firmicutes* dominated the microbial community. At the genus level, *Sediminibacterium* was the most abundant taxon, accounting for 23% of the total reads. Although *Sediminibacterium* species have not been directly demonstrated to oxidize Mn(II), this genus has been reported as a dominant member of microbial communities in Mn-rich biofiltration systems and sponge-based biofilms (Dangeti *et al.*, 2020; Aoki *et al.*, 2025), suggesting an ecological association with Mn-containing environments. Other genera with relative abundances exceeding 1% included *Ralstonia* (5.9%), *Sphingomonas* (2.9%), and *Rhodococcus* (1.2%). These genera were previously reported to include MnOB or Mn-associated taxa in engineered and environmental systems (Matsushita *et al.*, 2020; Jofré *et al.*, 2021; Zhu *et al.*, 2025). Overall, the enriched microbial community was dominated by taxa commonly reported as MnOB or Mn-associated taxa, consistent with the long-term operation of the reactor.

### Exploratory transcriptomic comparison of Mn(II)-oxidizing and Mn(IV)-reducing states under aerobic conditions

To obtain preliminary insights into the gene expression patterns associated with the Mn(II)-oxidizing and Mn(IV)-reducing states under aerobic conditions, biomass samples representing the two metabolic states of *P. resinovorans* MO-1 were subjected to RNA sequencing. Since biological replicates were not included in this exploratory anal­ysis, gene expression patterns were interpreted descriptively based on transcript abundance (TPM), and a statistical differential expression anal­ysis was not performed. A total of 5,751 genes were detected across the two states. Selected Mn oxidation-related genes, genes related to the respiratory chain, and a redox-related gene were plotted in Fig. 5 to visualize differences in transcript abundance between the‍ ‍Mn(II)-oxidizing and Mn(IV)-reducing states. These included MCOs, AHPs, catalase–peroxidase (*katG*), and flagellar assembly proteins, as well as genes encoding components of the respiratory chain, including NADH dehydrogenase (Complex I; *nuo*), succinate dehydrogenase (Complex II; *sdh*), ubiquinone biosynthesis proteins (*ubi*), the cytochrome bc_1_ complex (Complex III; *pet*), cbb_3_-type cytochrome c oxidase (Complex IV; *cco*), ATP synthase (Complex V; *atp*), and ferredoxin/flavodoxin–NADP^+^ reductase (*fpr*). Positive values indicate higher transcript abundance in the Mn(II)-oxidizing state, while negative values indicate higher transcript abundance in the Mn(IV)-reducing state. In addition, a descriptive overview of all detected genes and a heatmap of the selected genes are provided as supporting information (Fig. S3 and S4).

Genes associated with MCOs, AHPs, *katG*, and flagellar assembly generally showed higher transcript abundance in the Mn(II)-oxidizing state than in the Mn(IV)-reducing state. Among these groups, genes encoding MCOs, *katG*, and flagellar components showed higher transcript abundance in the Mn(II)-oxidizing state. These functional groups have been widely implicated in biological Mn oxidation (Geszvain *et al.*, 2016; Matsushita *et al.*, 2018b; Zhao *et al.*, 2023), and the expression patterns observed here are consistent with previous findings.

In contrast, under the Mn(IV)-reducing state, several genes involved in respiratory electron transfer generally showed higher transcript abundance. Genes encoding the full set of aerobic respiratory chain components were detected, including *nuo*, *sdh*, *ubi*, *pet*, *cco*, and *atp*. Notably, *nuo* genes showed higher transcript abundance in the Mn(IV)-reducing state, which may reflect greater electron flow through the respiratory chain under these conditions. In addition, *fpr*, which functions as an electron transfer protein accepting electrons from NADPH and transferring them to ferredoxin or flavodoxin in *Pseudomonas* (Yeom *et al.*, 2009), showed higher transcript abundance in the Mn(IV)-reducing state, consistent with changes in intracellular redox turnover.

Overall, this exploratory transcriptomic comparison revealed descriptive patterns that were broadly consistent with the distinct Mn redox phenotypes observed under the two conditions. The transcript abundance of Mn oxidation-related genes was generally higher in the Mn(II)-oxidizing state, while those of several respiratory electron transfer-related genes were typically higher in the Mn(IV)-reducing state. Although preliminary, these transcriptomic observations provide supportive evidence for physiological differences between the two Mn redox states.

## Discussion

The present study showed that Mn(II) oxidation rates were the highest at low organic substrate concentrations and declined as substrate concentrations increased in the reactor. The same pattern was observed in enriched-biomass and pure-culture batch experiments, showing that it was robust and not system-specific. These results align with previous findings demonstrating that MnOB are frequently associated with oligotrophic environments (Tebo *et al.*, 2005; Miyata *et al.*, 2024) and are preferentially enriched under low organic substrate concentrations (Cao *et al.*, 2015; Matsushita *et al.*, 2018a; Shoiful *et al.*, 2020), consistent with the view that low organic substrate concentrations favor Mn(II) oxidation activity. Importantly, the results of pure-culture batch experiments suggest that the suppression of Mn(II) oxidation and COD removal at elevated substrate concentrations reflect an intrinsic physiological constraint of MnOB rather than competitive exclusion by non–MnOB bacteria.

Although Liang *et al.* (2016) reported that nutrient levels markedly affected Mn(II) oxidation by *Sphingopyxis* sp. QXT-31, the effective substrate concentration range was not clearly defined. In contrast, the present study demonstrated that Mn(II) oxidation was favored at organic substrate concentrations below approximately 50 mg COD L^–1^, whereas concentrations around 100 mg COD L^–1^ were more favorable for MnOB growth. Accordingly, we propose that the pre-installation of MnO_2_ to suppress certain non-MnOB groups (Matsushita *et al.*, 2020), followed by the maintenance of organic substrate concentrations within 50–100 mg COD L^–1^, represents a practical operational strategy for effective MnOB enrichment and sustained Mn(II) oxidation in engineered systems.

COD removal rates sharply declined once the initial COD concentration exceeded 100 mg L^–1^ in both reactor and batch experiments (Fig. 3B and 4B), rather than stabilizing at higher substrate concentrations. This non-linear response suggests that elevated organic substrate concentrations constrain MnOB substrate utilization and growth. The observed pattern deviates from classical Michaelis–Menten kinetics and is characteristic of substrate inhibition. Such inhibitory behavior has been widely documented in ammonia-oxidizing and nitrite-oxidizing bacteria, where high substrate concentrations inhibit microbial growth and activity (Vadivelu *et al.*, 2006; Blackburne *et al.*, 2007). Substrate inhibition has also been reported in heterotrophic bacteria under high organic substrate conditions (Kayombo *et al.*, 2003; Amer and Kim, 2023). Previous studies primarily emphasized the inhibitory effects of elevated Mn(II) concentrations on the growth of MnOB (Therdkiattikul *et al.*, 2020); however, the inhibitory effects of organic substrates have not been systematically exami­ned. Therefore, the present results indicate that substrate inhibition occurs in MnOB at high organic substrate concentrations.

When organic substrate concentrations exceeded 1,000 mg COD L^–1^, Mn redox activity shifted toward net Mn(IV) reduction by *P. resinovorans* MO-1 under fully aerobic conditions. Biological Mn(IV) reduction has been associated with metal-reducing bacteria, such as *Geobacter* and *Shewanella*, under anaerobic conditions (Lovley *et al.*, 2004; Logan, 2009). In previous studies, Mn(IV) reduction after a prolonged incubation was often attributed to oxygen depletion (Cerrato *et al.*, 2010; Sujith *et al.*, 2014). In the present study, pure MnOB culture experiments supplemented with 5 mg Mn(II) L^–1^ and 3.3 g L^–1^ MnO_2_ were conducted under continuous aeration while maintaining the biomass in suspension, thereby minimizing the potential oxygen limitation associated with biofilm formation or mass transfer constraints. Moreover, COD removal remained low under Mn(IV) reduction conditions (Fig. 4), indicating that overall organic substrate utilization was not markedly enhanced. Despite these fully aerobic conditions, a reversal in the net Mn redox direction—from net Mn(II) oxidation to net Mn(IV) reduction—was observed once organic substrate concentrations exceeded 1,000 mg COD L^–1^. This threshold response shows that high organic substrate concentrations may induce Mn(IV) reduction in *P. resinovorans* MO-1, allowing MnO_2_ to function as an electron sink even in the presence of oxygen. Collectively, these results demonstrate that aerobic Mn(IV) reduction may occur at high organic substrate concentrations.

To obtain preliminary insights into the physiological shift from Mn(II) oxidation to Mn(IV) reduction under different organic substrate concentrations, we performed an exploratory transcriptomic comparison of *P. resinovorans* MO-1 under Mn(II)-oxidizing and Mn(IV)-reducing states. Under the Mn(II)-oxidizing state, canonical Mn(II) oxidation-associated genes, including those encoding MCOs, *katG*, and flagellar-related functions, generally showed higher transcript abundance. This pattern was broadly consistent with the batch results, which showed that Mn(II) oxidation was kinetically favored at low organic substrate concentrations. Notably, these genes were still detected under the Mn(IV)-reducing state, indicating that Mn(II) oxidation-related functions were not completely absent under high organic substrate conditions. This interpretation is also consistent with detectable Mn(II) oxidation observed at 1,000 mg COD L^–1^ in the reactor (Fig. 3A).

The higher transcript abundances of *nuo* and *fpr* were observed during the Mn(IV)-reducing state. The *nuo* genes encode NADH dehydrogenase (Complex I), which catalyzes initial electron entry from NADH into the respiratory chain (Ciemniecki and Newman, 2023). Accordingly, higher *nuo* transcript abundance may reflect greater electron flow through the respiratory network under these conditions. Higher *fpr* transcript abundance was also observed, and *fpr* is known to mediate electron transfer between NADPH and ferredoxin and to contribute to intracellular redox homeostasis (Spaans *et al.*, 2015). Although overall COD removal did not increase at high organic substrate concentrations, the combined increase in *nuo* and *fpr* transcript abundances was consistent with changes in intracellular redox turnover under carbon-rich conditions. Furthermore, Hirose *et al.* (2018) demonstrated the flow of electrons from NADH through the respiratory chain to MnO_2_ in the Mn-reducing bacterium *Shewanella oneidensis* MR-1. While this mechanism has not been demonstrated in *P. resinovorans* MO-1, the observed transcriptomic pattern is consistent with high organic substrate concentrations potentially favoring conditions under which a portion of the intracellular reducing power is associated with Mn(IV) reduction in addition to aerobic respiration. However, this interpretation remains preliminary, and the mechanisms underlying aerobic Mn(IV) reduction in *P. resinovorans* MO-1 have yet to be elucidated in detail.

Overall, low organic substrate conditions were associated with stronger Mn(II) oxidation activity, which was accompanied by the higher transcript abundance of selected Mn(II) oxidation-related genes. In contrast, high organic substrate conditions were associated with Mn(IV) reduction, which may be related to changes in respiratory electron transfer and intracellular redox-related processes. However, this transcriptomic comparison was limited because it included only two conditions and no biological replicates. Therefore, the transcript abundance patterns observed cannot clearly distinguish the effects of organic substrate concentrations from those of the Mn redox state. Accordingly, the transcriptomic results obtained herein need to be interpreted as supportive information for understanding the observed Mn redox changes, and further studies with appropriate control conditions are needed to clarify the mechanisms underlying aerobic Mn(IV) reduction in more detail.

## Conclusion

The present study showed that organic substrate concentrations were a critical operational parameter controlling Mn redox behavior in MnOB. Low organic substrate concentrations (50–100 mg COD L^–1^) favored the stable enrichment of MnOB and efficient Mn(II) oxidation, while excessive organic substrate concentrations suppressed Mn(II) oxidation and induced net Mn(IV) reduction even under aerobic conditions. These results identify an operational concentration window for maintaining Mn oxidation performance and highlight the importance of controlling organic substrate concentrations for the stable operation of biological Mn oxidation processes in engineered systems. Although the broader applicability of this response across diverse MnOB has yet to be confirmed, the present results provide a useful basis for process optimization and reactor operation. Further studies are needed to clarify the mechanisms underlying the transition between Mn oxidation and Mn reduction under different organic substrate concentrations.

## Citation

Chen, Y., Yoshimura, M., Kambara, H., Shoiful, A., Matsushita, S., Kindaichi, T., et al. (2026) Effects of Organic Substrate Concentrations on Biological Mn Oxidation and Reduction under Aerobic Conditions. *Microbes Environ ***41**: ME26046.

https://doi.org/10.1264/jsme2.ME26046

## Supplementary Material

Supplementary Material

## Figures and Tables

**Fig. 1.**
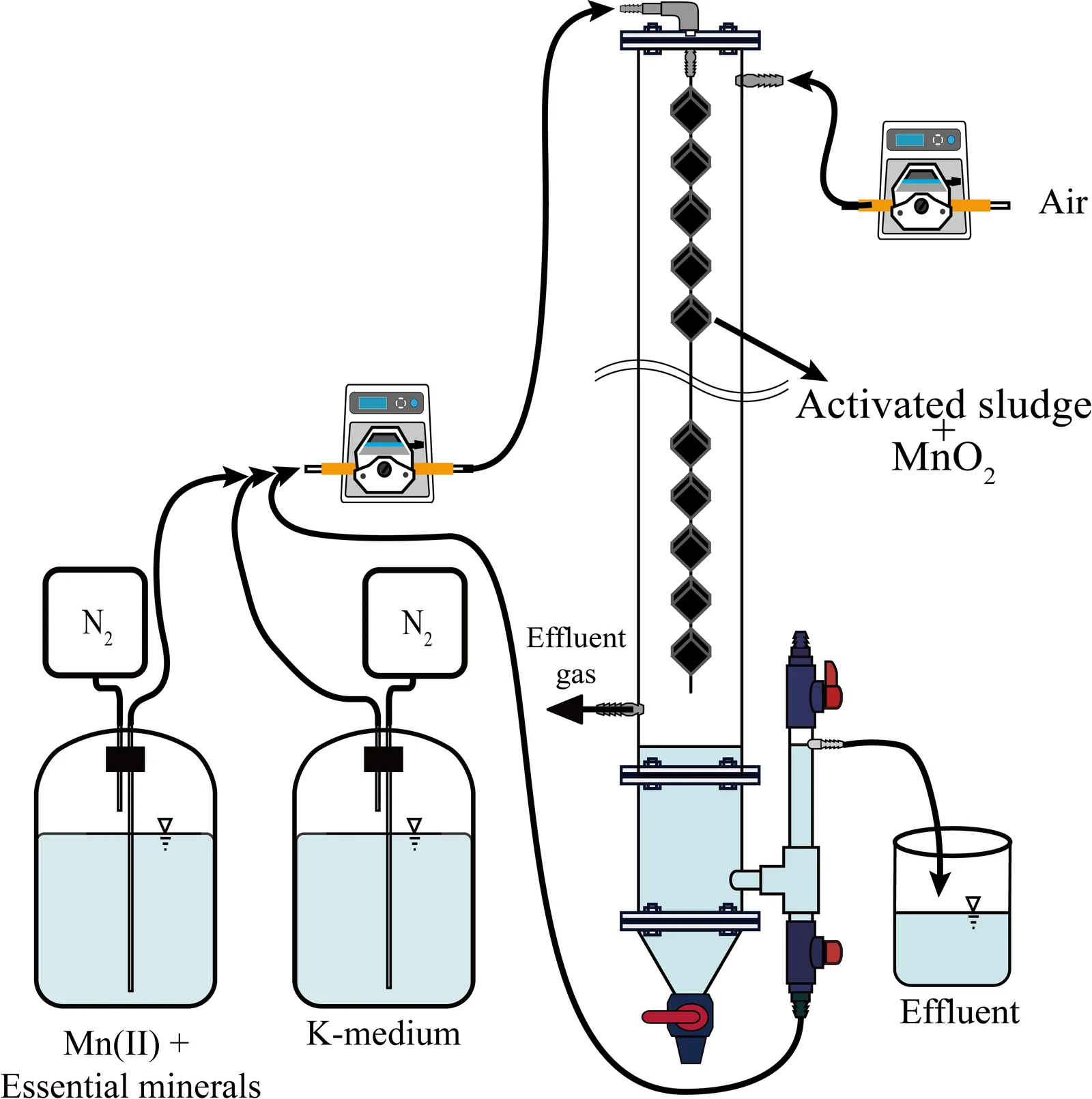
Configuration of the down-flow hanging sponge (DHS) system.

**Fig. 2.**
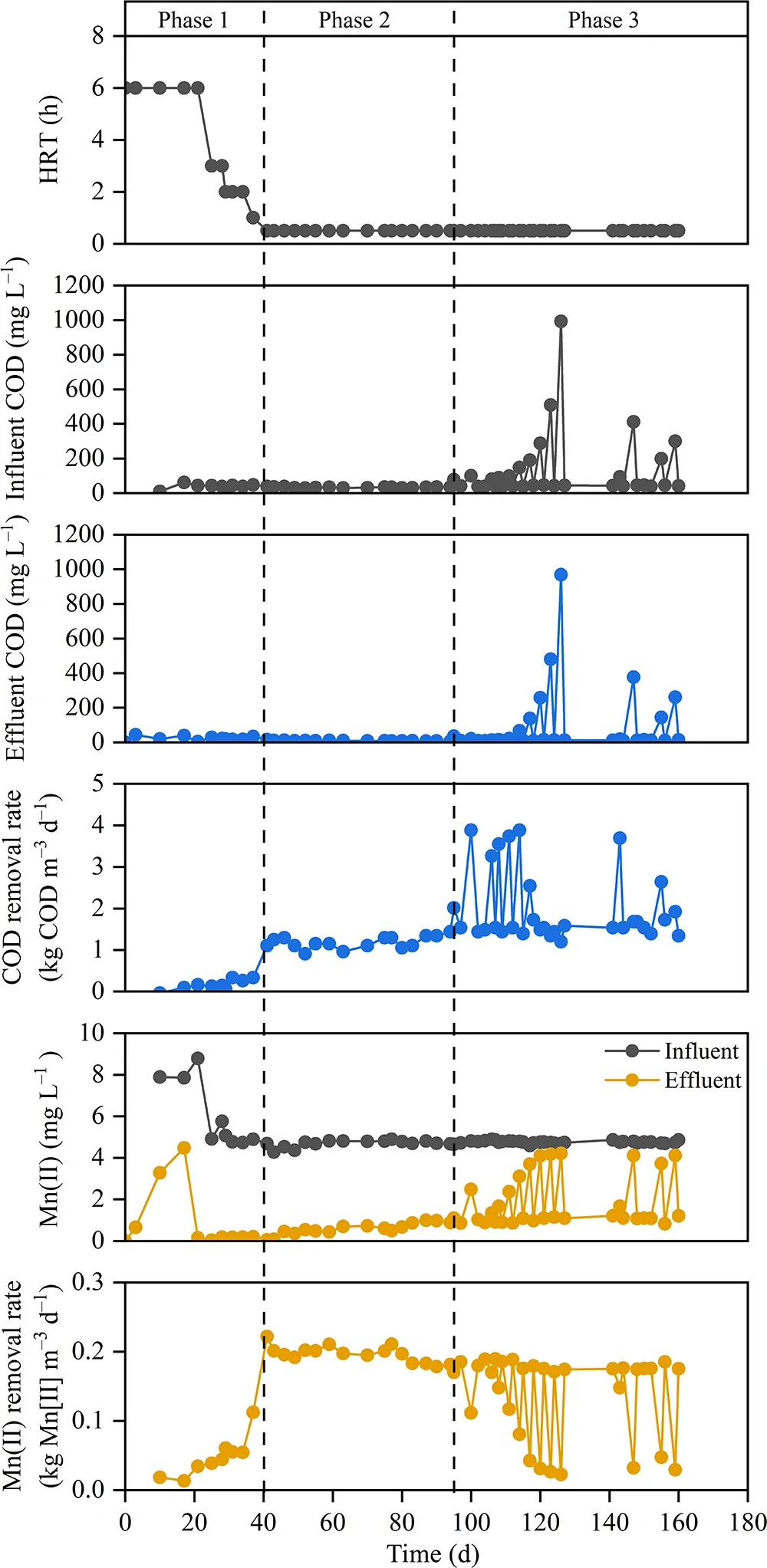
Performance of the DHS reactor.

**Fig. 3.**
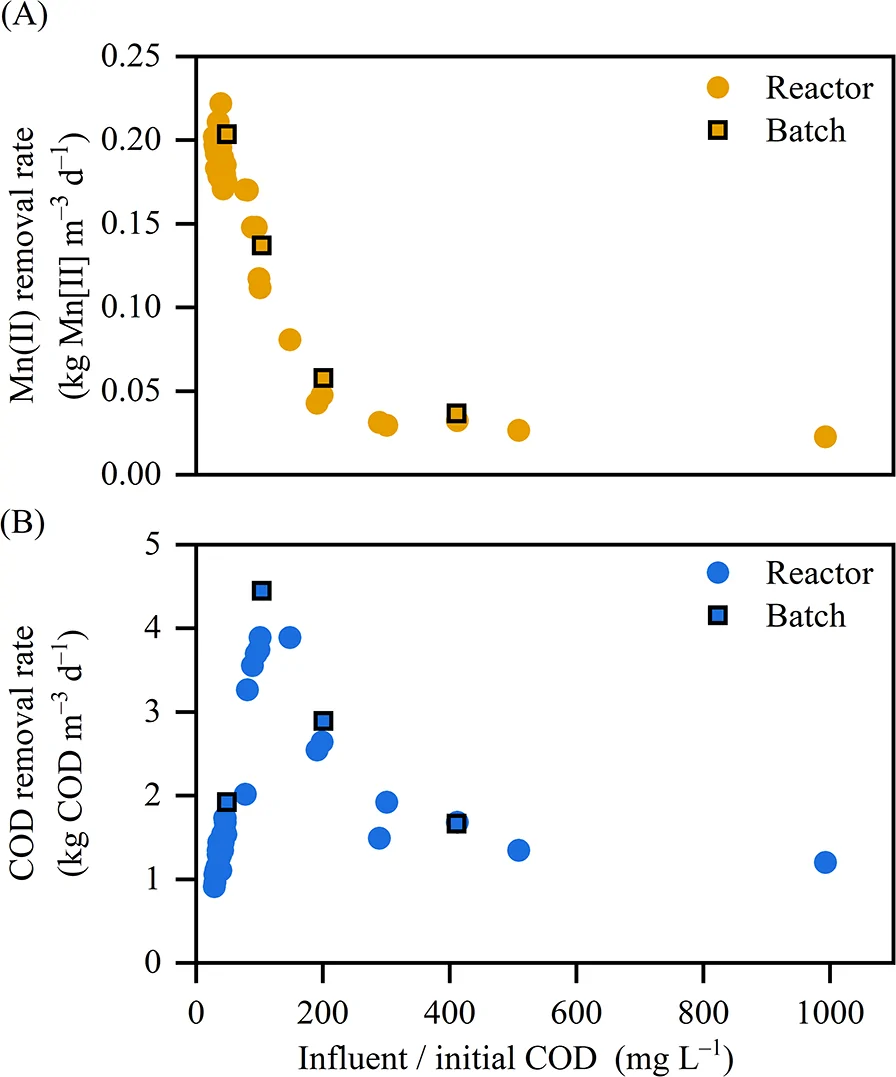
Relationship between the concentration of COD and (A) the Mn(II) removal rate and (B) COD removal rate in the reactor (Phases 2 and 3) and batch experiments with reactor-enriched biomass. In the reactor, COD refers to influent COD, while in batch experiments, COD refers to initial COD.

**Fig. 4.**
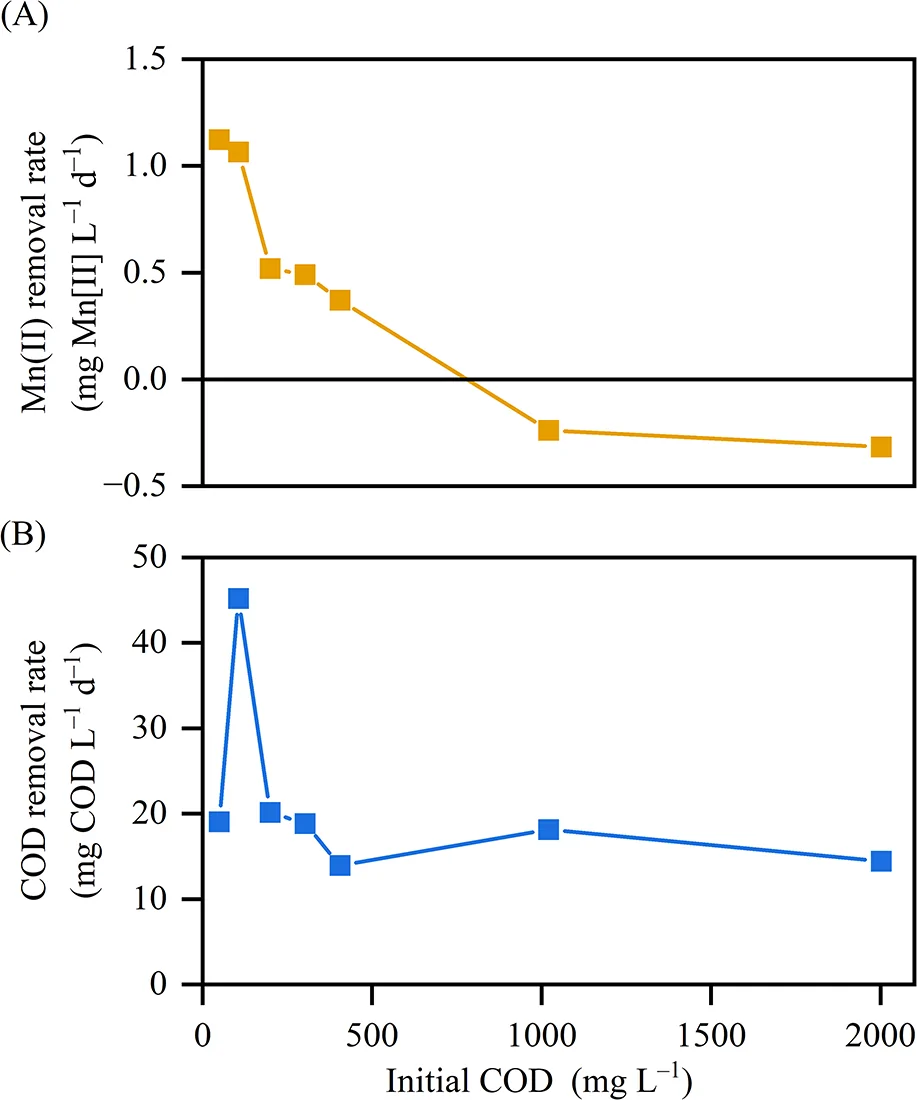
Relationships between the initial COD concentration and (A) the Mn(II) removal rate and (B) COD removal rate in batch experiments with *Pseudomonas resinovorans* MO-1.

**Fig. 5.**
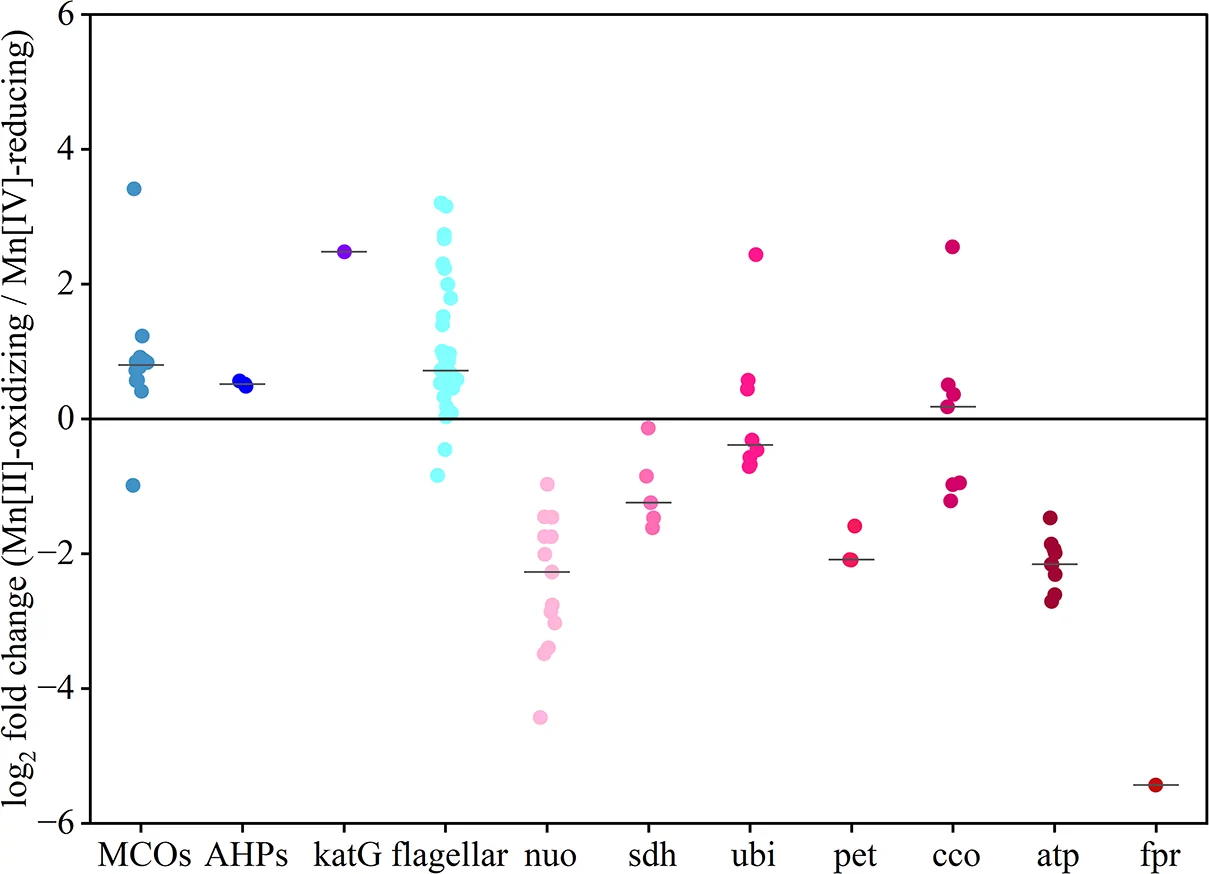
Comparison of selected gene expression between Mn(II)-oxidizing and Mn(IV)-reducing states in *Pseudomonas resinovorans* MO-1 under aerobic conditions. Each point represents an individual gene, and horizontal bars indicate the median value for each gene group. The y-axis shows the log_2_ fold change calculated as log_2_ (TPM in the Mn[II]-oxidizing state/TPM in the Mn[IV]-reducing state). Selected gene groups include multicopper oxidases (MCOs), animal heme peroxidases (AHPs), catalase-peroxidase (katG), flagellar genes, genes related to the respiratory chain, and the redox-related gene fpr.

**Table 1.** Operational conditions of the DHS reactor.

Phase	Day	COD (mg L^–1^)	Mn(II) (mg L^–1^)	HRT (h)	pH	Supplied gas
1	0–40	10–62	5–9	0.5–6	6.4–8.1	Air
2	41–94	29–39	5	0.5	6.8–7.8	Air
3	95–160	36–993	5	0.5	6.8–7.4	O_2_
