# Supplementary material for: Effects of Organic Substrate Concentrations on Biological Mn Oxidation and Reduction under Aerobic Conditions
Source: Microbes Environ. 2026 Sep 12;41(3):ME26046. doi: 10.1264/jsme2.ME26046 (PMC13659340; doi:10.1264/jsme2.ME26046)
Supplement: Supplementary file 1 [file 41_26046_s1.pdf]

Supplementary Materials

For

Effects of Organic Substrate Concentrations on Biological Mn Oxidation and Reduction under Aerobic Conditions

Yangbo Chen[1*], Mizuki Yoshimura[1], Hiromi Kambara[2], Ahmad Shoiful[3], Shuji Matsushita[4], Tomonori Kindaichi[1], Yoshiteru Aoi[5], and Akiyoshi Ohashi[1*]

Supplementary materials list:

Supplementary Information

1. Substrate composition
2. Analytical methods
3. Microbial community analysis
4. Transcriptomic analysis of *P. resinovorans* MO-1

Fig. S1. Changes in Mn(II) and COD concentrations during batch experiments using enriched biomass collected from the reactor at initial COD concentrations of (A) 50, (B) 100, (C) 200, and (D) 400 mg L^{-1}. For all batch tests, the biomass concentration was diluted 50-fold relative to that in the reactor sponges. Linear regression lines are shown for Mn(II) and COD to calculate removal rates.

Fig. S2. Changes in Mn(II) and COD concentrations during batch experiments with *P. resinovorans* strain MO-1 at initial COD concentrations of (A) 50, (B) 100, (C) 200, (D)

300, (E) 400, (F) 1,000, and (G) 2,000 mg L^{-1}. Linear regression lines are shown for Mn(II) and COD to calculate removal or production rates.

Fig. S3. Genome-wide MA plot of transcript abundance differences between Mn(II)-oxidizing and Mn(IV)-reducing states in *P. resinovorans* MO-1. Each point represents an individual gene. The x-axis shows the mean expression level based on $\log_2$ TPM, and the y-axis shows the $\log_2$ fold change calculated as Mn(II)-oxidizing state / Mn(IV)-reducing state. Positive values indicate higher transcript abundance in the Mn(II)-oxidizing state, whereas negative values indicate higher transcript abundance in the Mn(IV)-reducing state. Colored symbols represent selected functional gene groups: MCOs, multicopper oxidases; AHPs, animal heme peroxidases; *katG*, catalase–peroxidase; flagellar, flagellar assembly proteins; *nuo*, Complex I NADH dehydrogenase subunits (*nuoA*, *nuoB*, *nuoC*, *nuoE*, *nuoF*, *nuoG*, *nuoH*, *nuoI*, *nuoJ*, *nuoK*, *nuoL*, *nuoM*, and *nuoN*); *sdh*, Complex II succinate dehydrogenase (*sdhA–D*); *ubi*, ubiquinone biosynthesis proteins (*ubiA*, *ubiB*, *ubiC*, *ubiD*, *ubiE*, *ubiG*, *ubiH*, and *ubiX*); *pet*, Complex III cytochrome bc_1 complex (*petA–C*); *cco*, Complex IV cbb_3-type cytochrome c oxidase (*ccoN*, *ccoO*, and *ccoP*); *atp*, Complex V ATP synthase subunits (*atpA–I*); *fpr*, ferredoxin/flavodoxin–$NADP^+$ reductase.

Fig. S4. Heatmap of transcript abundance for selected Mn oxidation-related genes, genes related to the respiratory chain, and the redox-related gene in *P. resinovorans* MO-1. The two columns represent the Mn(II)-oxidizing and Mn(IV)-reducing states. Color intensity indicates $\log_2$ TPM values. Gene groups include MCOs, AHPs, *katG*, flagellar assembly genes, genes related to the respiratory chain (*nuo*, *sdh*, *ubi*, *pet*, *cco*, and *atp*), and the redox-related gene *fpr*.

Table S1. Most dominant genera in the reactor with relative abundances ≥1% of total sequence reads.

Supplementary Information

1. Substrate composition

Mn(II) was supplied at 5 mg L^{-1} using $MnCl_2{\cdot}4H_2O$, and the mineral medium contained $FeSO_4{\cdot}7H_2O$ (0.1 mg L^{-1}), Na_2HPO_4 (20 mg L^{-1}), $CaCl_2{\cdot}2H_2O$ (0.05 mg L^{-1}), $MgSO_4{\cdot}7H_2O$ (0.2 mg L^{-1}), $CuSO_4{\cdot}5H_2O$ (0.0025 mg L^{-1}), Na_2SeO_4 (0.005 mg L^{-1}), $NiCl_2{\cdot}6H_2O$ (0.019 mg L^{-1}), $CoCl_2{\cdot}6H_2O$ (0.024 mg L^{-1}), $Na_2MoO_4{\cdot}2H_2O$ (0.022 mg L^{-1}), H_3BO_3 (0.001 mg L^{-1}), and $ZnSO_4$ (0.043 mg L^{-1}).

2. Analytical methods

Influent and effluent samples were collected periodically and filtered through 0.45 μm PTFE membrane filters (Advantec Co., Ltd., Tokyo, Japan). Dissolved Mn(II) and chemical oxygen demand (COD) were measured using a DR-2800 spectrophotometer (Hach, Loveland, CO, USA) following the manufacturer's standard methods.

3. Microbial community analysis

The biomass samples were collected on day 160 by squeezing all sponge cubes after completion of reactor operation. Genomic DNA was extracted using the FastDNA SPIN Kit for Soil (MP Biomedicals, USA). The extracted DNA was sent to the Bioengineering Laboratory (Kanagawa, Japan) for amplification of the V3–V4 hypervariable region of the 16S rRNA gene using primers 341f (5′-CCTACGGGNGGCWGCAG-3′) and 805r (5′-GACTACHVGGGTATCTAATCC-3′). Amplicon sequencing was performed on an Illumina MiSeq platform (2 × 300 bp paired-end; MiSeq Reagent Kit v3). Sequence processing, including primer trimming and quality filtering, was conducted as described by Shoiful *et al*. (2024).

4. Transcriptomic analysis of *P. resinovorans* MO-1

Two distinct Mn redox states were established for gene expression analysis. Under the Mn(II)-oxidizing state, the organic substrate concentration was adjusted to 200 mg COD L^{-1} with Mn(II) supplied at 5 mg L^{-1}. Under the Mn(IV)-reducing state, the organic substrate concentration was adjusted to 3,000 mg COD L^{-1} with Mn(II) supplied at 5 mg L^{-1}, and MnO_2 was additionally added at 2 g L^{-1}. Each 122 mL vial contained 80 mL of substrate prepared under the respective redox state and was inoculated with biomass harvested at $OD_{600} \approx 0.5$. The cultures were incubated under aerobic conditions for 3 days.

After incubation, 45 mL of culture was centrifuged at 5,000 rpm for 5 min. The supernatant was discarded, and the pellet was resuspended in 500 µL of residual liquid and transferred to a 2 mL microcentrifuge tube containing beads. The suspension was centrifuged at 13,000 rpm for 1 min, and the supernatant was removed. Pellets were immediately frozen in liquid nitrogen and disrupted using a bead beater (3,200 rpm, 15 s), followed by refreezing; this freeze–disruption cycle was repeated twice. Subsequently, 500 µL of ISOGEN reagent was added, and total RNA was extracted according to the manufacturer's protocol (ISOGEN with Column). Purified RNA was dissolved in 25 µL TE buffer.

The extracted RNA samples were sent to the Bioengineering Laboratory (Japan) for bacterial rRNA removal and directional cDNA library preparation using the MGIEasy RNA Directional Library Prep Set (MGI Tech Co., Ltd.) according to the manufacturer's instructions. DNA nanoballs (DNBs) were generated, and sequencing was performed on a DNBSEQ-G400 platform (MGI Tech Co., Ltd.) with 2 × 100 bp

paired-end reads. Raw reads were adapter-trimmed and quality-filtered using Trimmomatic (v0.39) to remove bases with Phred scores < 20 and paired reads shorter than 40 bp (Bolger *et al*., 2014). The filtered reads were aligned to the reference genome of *P. resinovorans* MO-1 (GCF_002003485.1) using HISAT2 (v2.2.1). The resulting alignment files were processed with SAMtools, and transcript abundance was quantified using StringTie (v2.1.7). Functional annotation of the quantified transcripts was performed using eggNOG-mapper (v2.1.11) (Cantalapiedra *et al*., 2021).

Bolger, A.M., Lohse, M., Usadel, B., 2014. Trimmomatic: A flexible trimmer for Illumina sequence data. Bioinformatics 30, 2114–2120. https://doi.org/10.1093/bioinformatics/btu170

Cantalapiedra, C.P., Hernández-Plaza, A., Letunic, I., Bork, P., Huerta-Cepas, J., 2021. eggNOG-mapper v2: Functional Annotation, Orthology Assignments, and Domain Prediction at the Metagenomic Scale. Mol. Biol. Evol. 38, 5825–5829. https://doi.org/10.1093/molbev/msab293

Shoiful, A., Ohta, T., Kambara, H., Matsushita, S., Kindaichi, T., Ozaki, N., Aoi, Y., Ohashi, A., 2024. Inhibition of Mn(II) oxidation performance in the presence of high residual Mn(II) concentrations. Biochem. Eng. J. 208, 109373. https://doi.org/10.1016/J.BEJ.2024.109373

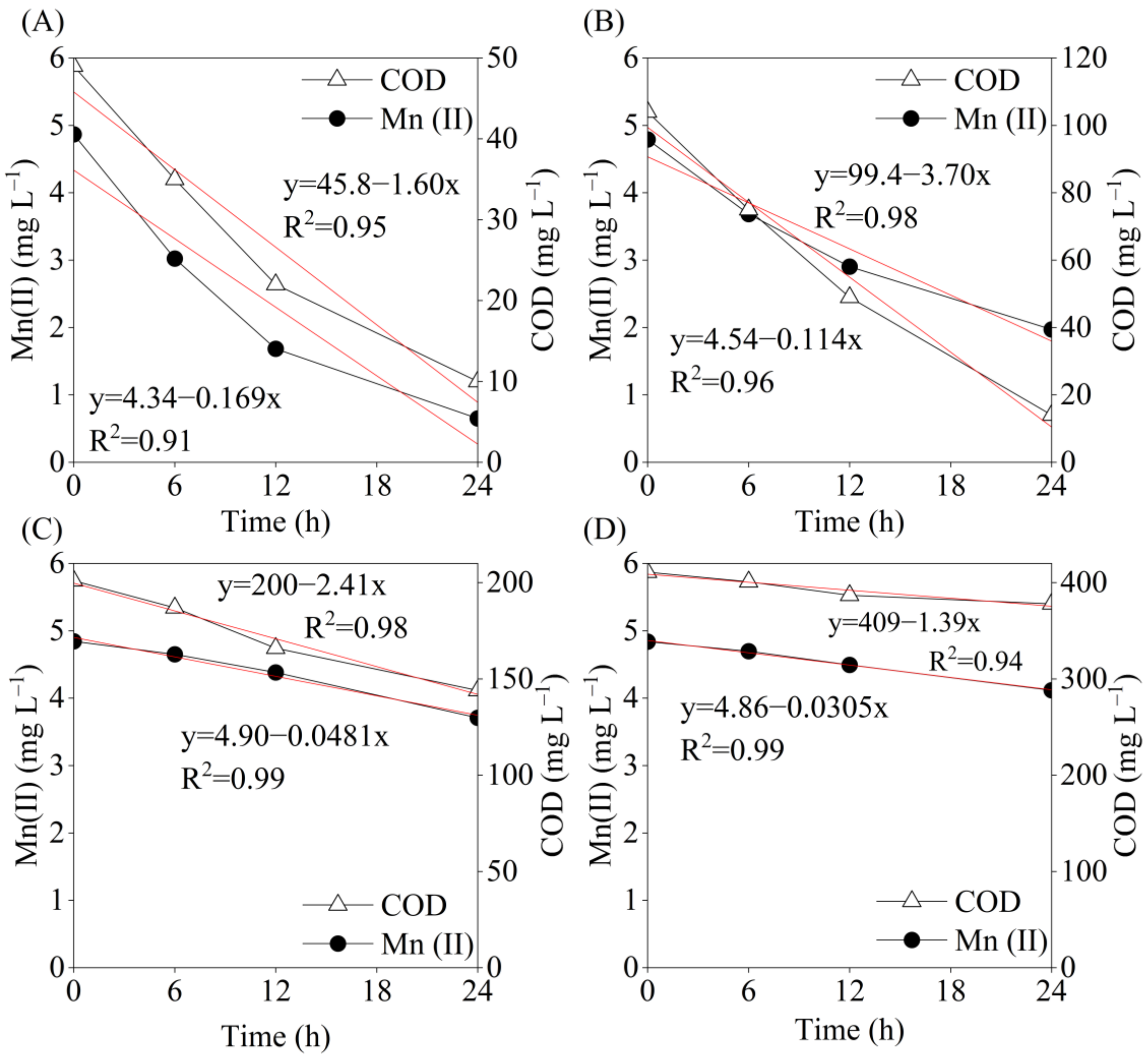

Fig. S1. Changes in Mn(II) and COD concentrations during batch experiments using enriched biomass collected from the reactor at initial COD concentrations of (A) 50, (B) 100, (C) 200, and (D) 400 mg L^{-1}. For all batch tests, the biomass concentration was diluted 50-fold relative to that in the reactor sponges. Linear regression lines are shown for Mn(II) and COD to calculate removal rates.

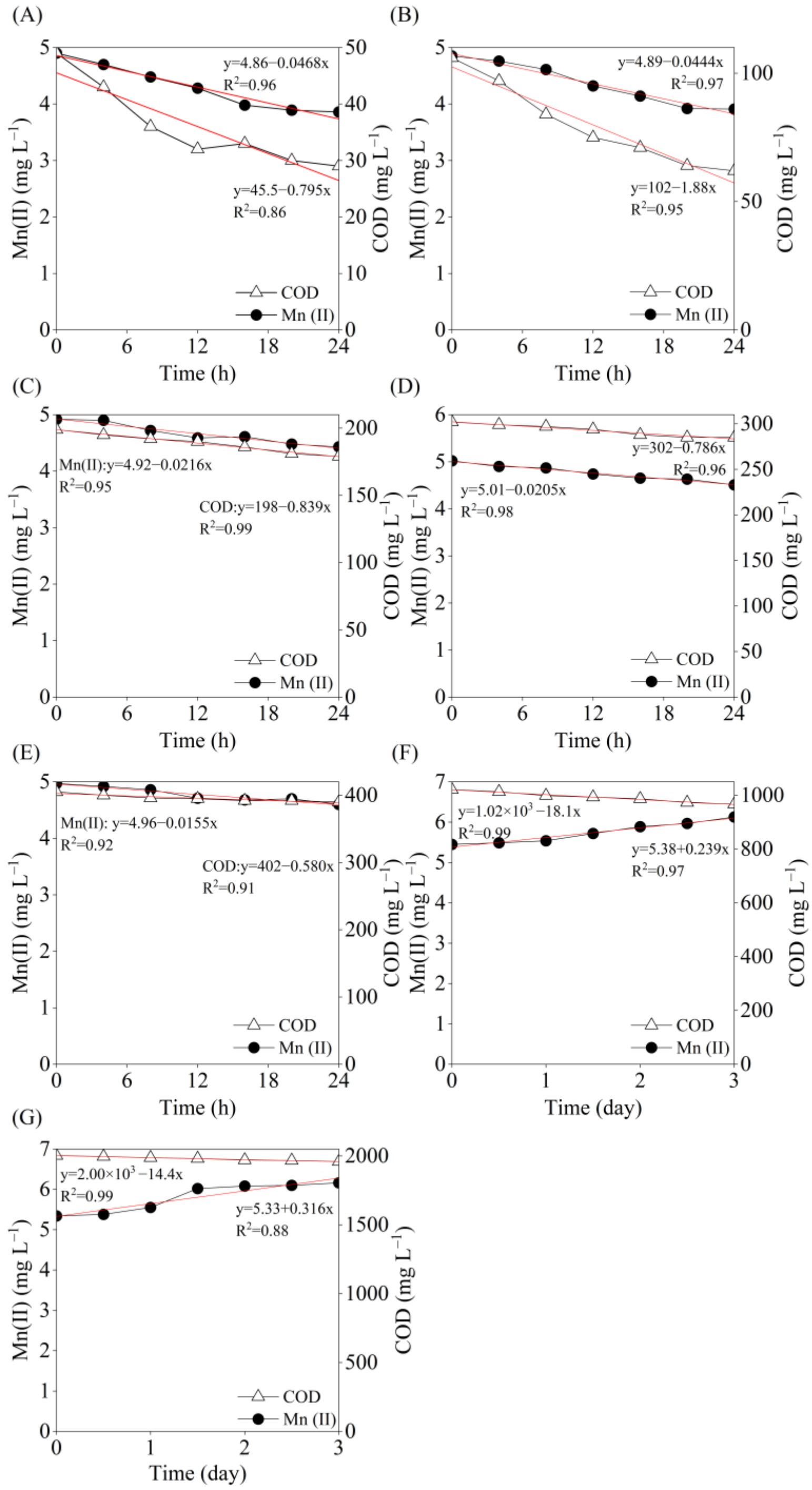

Fig. S2. Changes in Mn(II) and COD concentrations during batch experiments with *P. resinovorans* strain MO-1 at initial COD concentrations of (A) 50, (B) 100, (C) 200, (D) 300, (E) 400, (F) 1,000, and (G) 2,000 mg L^{-1}. Linear regression lines are shown for Mn(II) and COD to calculate removal or production rates.

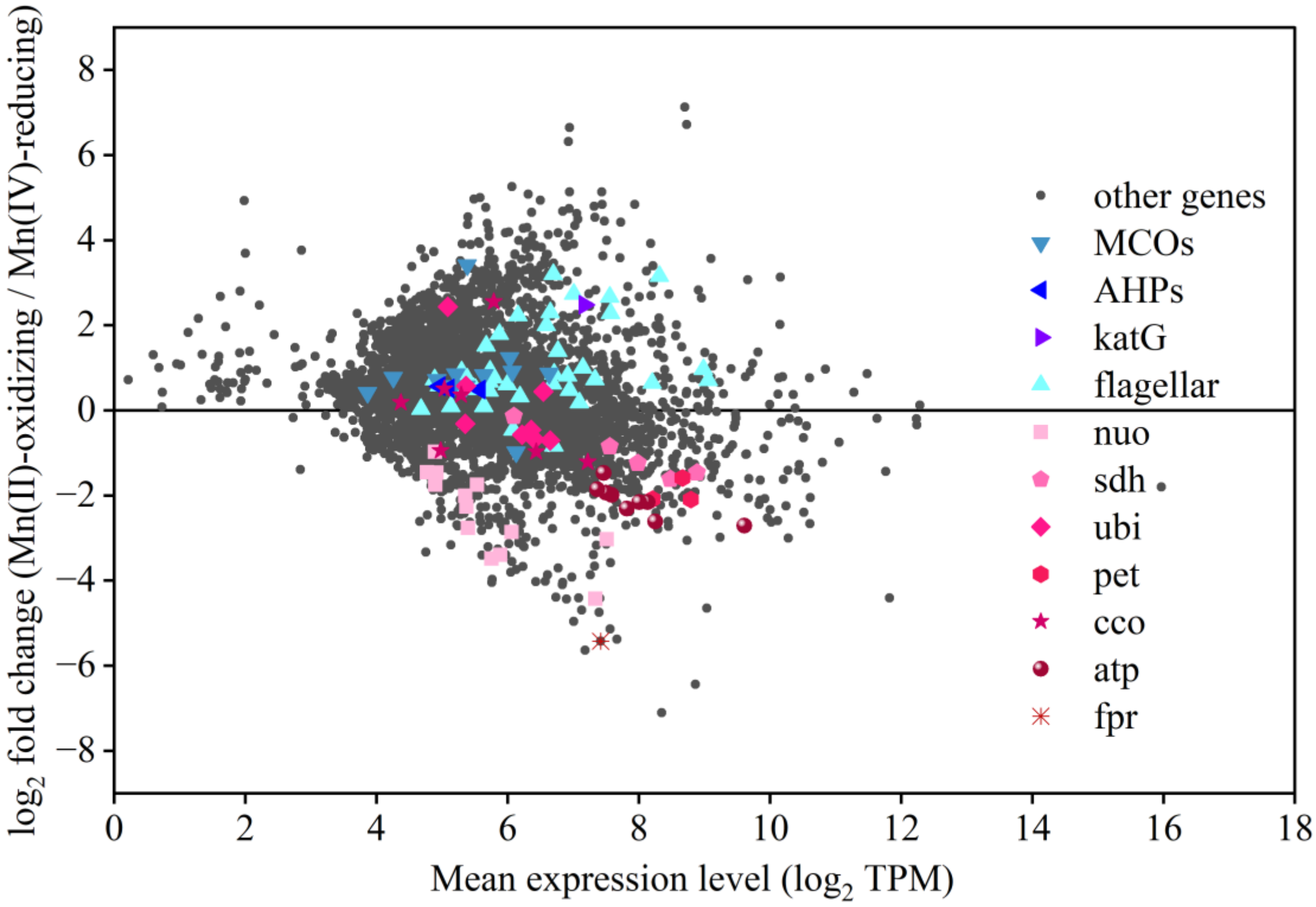

Fig. S3. Genome-wide MA plot of transcript abundance differences between Mn(II)-oxidizing and Mn(IV)-reducing states in *P. resinovorans* MO-1. Each point represents an individual gene. The x-axis shows the mean expression level based on $\log_2$ TPM, and the y-axis shows the $\log_2$ fold change calculated as $\log_2$(TPM in the Mn(II)-oxidizing state / TPM in the Mn(IV)-reducing state). Positive values indicate higher transcript abundance in the Mn(II)-oxidizing state, whereas negative values indicate higher transcript abundance in the Mn(IV)-reducing state. Colored symbols represent selected functional gene groups: MCOs, multicopper oxidases; AHPs, animal heme peroxidases; *katG*, catalase–peroxidase; flagellar, flagellar assembly proteins; *nuo*, Complex I NADH dehydrogenase subunits (*nuoA*, *nuoB*, *nuoC*, *nuoE*, *nuoF*, *nuoG*, *nuoH*, *nuoI*, *nuoJ*, *nuoK*, *nuoL*, *nuoM*, and *nuoN*); *sdh*, Complex II succinate dehydrogenase (*sdhA–D*); *ubi*, ubiquinone biosynthesis proteins (*ubiA*, *ubiB*, *ubiC*, *ubiD*, *ubiE*, *ubiG*, *ubiH*, and *ubiX*); *pet*, Complex III cytochrome bc_1 complex (*petA–C*); *cco*, Complex IV cbb_3-type cytochrome c oxidase (*ccoN*, *ccoO*, and *ccoP*); *atp*, Complex V ATP synthase subunits (*atpA–I*); *fpr*, ferredoxin/flavodoxin–$NADP^+$ reductase.

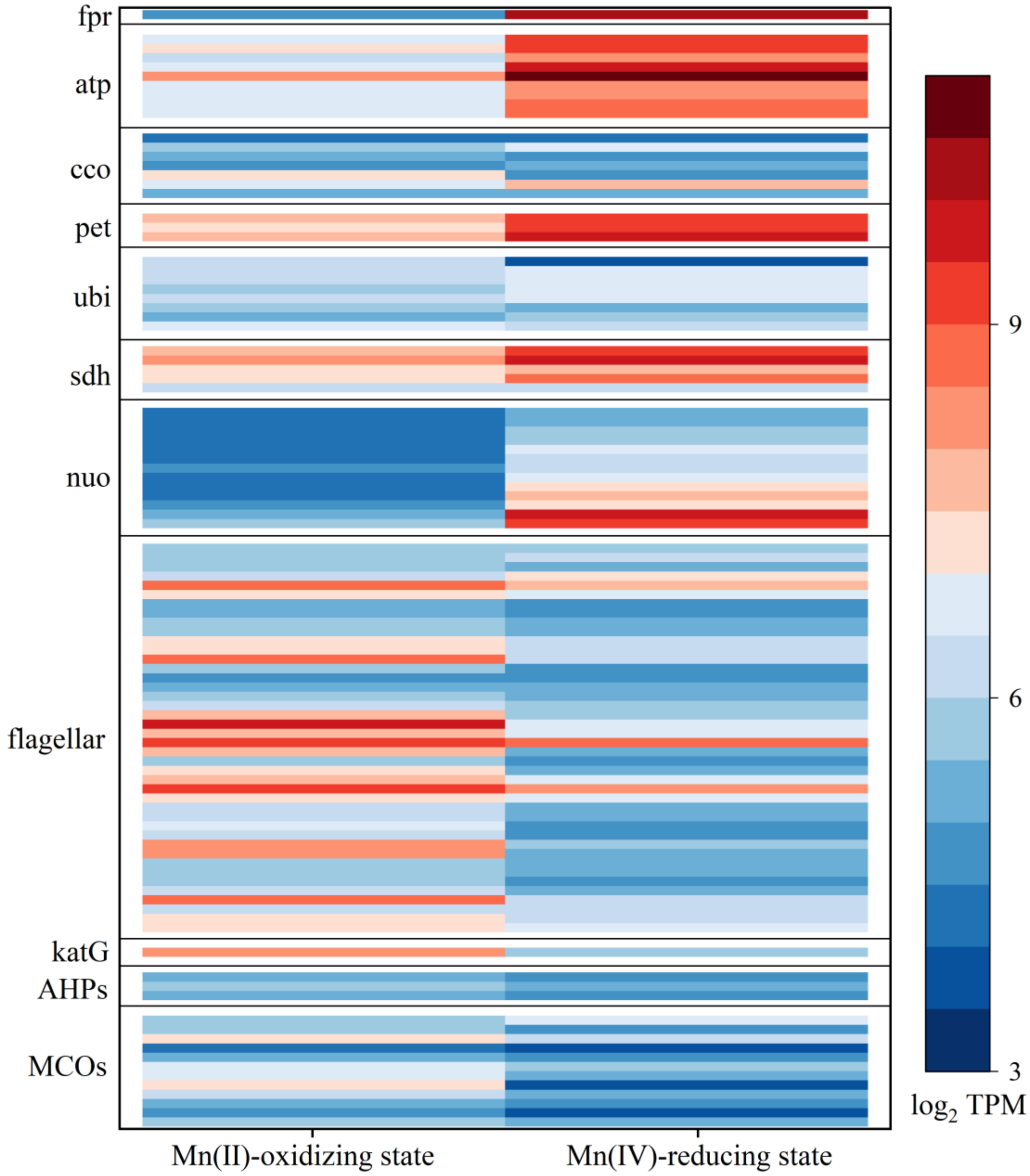

Fig. S4. Heatmap of transcript abundance for selected Mn oxidation-related genes, genes related to the respiratory chain, and the redox-related gene in *P. resinovorans* MO-1. The two columns represent the Mn(II)-oxidizing and Mn(IV)-reducing states. Color intensity indicates $\log_2$ TPM values. Gene groups include MCOs, AHPs, *katG*, flagellar assembly genes, genes related to the respiratory chain (*nuo*, *sdh*, *ubi*, *pet*, *cco*, and *atp*), and the redox-related gene *fpr*.

Table S1. Most dominant genera in the reactor with relative abundances ≥1% of total sequence reads.

Phylum	Class	Order	Family	Genus	Relative abundance (%)
Actinobacteria	*Actinobacteria*	*Actinomycetales*	*Nocardiaceae*	*Rhodococcus**	1.2
	Solibacteres	*Solibacterales*	unclassified	unclassified	2.8
Bacteroidetes	*Saprospirae*	*Saprospirales*	*Chitinophagaceae*	*Sediminibacterium*	23
Chlamydiae	*Chlamydiia*	*Chlamydiales*	*Parachlamydiaceae*	unclassified	2.8
Firmicutes	*Clostridia*	*Clostridiales*	*Clostridiaceae*	*Clostridium*	4.5
			Veillonellaceae	G07	1.7
				Sporomusa	4.8
				unclassified	3.8
			unclassified	unclassified	2.1
Proteobacteria	*Alphaproteobacteria*	*Caulobacterales*	*Caulobacteraceae*	*Phenylobacterium*	1.0
				Caulobacter	1.0
				unclassified	5.4
		Rhizobiales	*Hyphomicrobiaceae*	*Rhodoplanes*	1.6
		Rhodospirillales	*Rhodospirillaceae*	*Phaeospirillum*	4.4
				unclassified	1.5
		Sphingomonadales	*Sphingomonadaceae*	*Sphingomonas**	2.9
		unclassified	unclassified	unclassified	2.2
	Betaproteobacteria	*Burkholderiales*	*Comamonadaceae*	*Aquabacterium*	2.4
			Oxalobacteraceae	*Ralstonia**	5.9
		Rhodocyclales	*Rhodocyclaceae*	*Azospira*	3.5
	Gammaproteobacteria	*Enterobacteriales*	*Enterobacteriaceae*	unclassified	2.3
Others					19.2

* Potential MnOB genera
